# Cerebral Degeneration in Amyotrophic Lateral Sclerosis Revealed by 3-Dimensional Texture Analysis

**DOI:** 10.3389/fnins.2016.00120

**Published:** 2016-03-30

**Authors:** Rouzbeh Maani, Yee-Hong Yang, Derek Emery, Sanjay Kalra

**Affiliations:** ^1^Department of Computing Science, University of AlbertaEdmonton, AB, Canada; ^2^Department of Radiology and Diagnostic Imaging, University of AlbertaEdmonton, AB, Canada; ^3^Departments of Medicine, Computing Science, and Biomedical Engineering, University of AlbertaEdmonton, AB, Canada

**Keywords:** amyotrophic lateral sclerosis, cerebral degeneration, texture analysis, MRI, biomarker

## Abstract

**Introduction:** Routine MR images do not consistently reveal pathological changes in the brain in ALS. Texture analysis, a method to quantitate voxel intensities and their patterns and interrelationships, can detect changes in images not apparent to the naked eye. Our objective was to evaluate cerebral degeneration in ALS using 3-dimensional texture analysis of MR images of the brain.

**Methods:** In a case-control design, voxel-based texture analysis was performed on T1-weighted MR images of 20 healthy subjects and 19 patients with ALS. Four texture features, namely, autocorrelation, sum of squares variance, sum average, and sum variance were computed. Texture features were compared between the groups by statistical parametric mapping and correlated with clinical measures of disability and upper motor neuron dysfunction.

**Results:** Texture features were different in ALS in motor regions including the precentral gyrus and corticospinal tracts. To a lesser extent, changes were also found in the thalamus, cingulate gyrus, and temporal lobe. Texture features in the precentral gyrus correlated with disease duration, and in the corticospinal tract they correlated with finger tapping speed.

**Conclusions:** Changes in MR image textures are present in motor and non-motor regions in ALS and correlate with clinical features. Whole brain texture analysis has potential in providing biomarkers of cerebral degeneration in ALS.

## Introduction

Amyotrophic lateral sclerosis (ALS) is a progressive degenerative disorder of adulthood leading to rapid accrual of muscle weakness and disability. The clinical features are secondary to degeneration of both upper motor neurons (UMN) of the cerebral cortex and lower motor neurons (LMN) in the brainstem and spinal cord. Cerebral degeneration exists beyond the motor cortex with frontotemporal degeneration being the substrate for cognitive impairment in upwards of 50% of patients. While the presence of LMN loss is supported by electromyography, an objective measure of UMN damage is lacking. A quantitative measure of cerebral degeneration is needed to aid diagnosis, evaluate novel therapies, and further our understanding of pathogenesis and the biological basis of the marked phenotypic heterogeneity observed in this complex disorder.

MRI holds promise in this regard to non-invasively quantify cerebral degeneration. Early MRI studies in ALS reported hyperintensity of the corticospinal tract (CST) on T2-weighted, (Goodin et al., 1988; Cheung et al., 1995) proton density, (Cheung et al., 1995; Waragai, 1997) and FLAIR, (Hecht et al., 2002) sequences and hypointensity of the posterior bank of the precentral gyrus on T2-weighted (Cheung et al., 1995) and FLAIR images (Hecht et al., 2002). However, the increased intensity of the CST in ALS is bilateral and symmetric relative to the normal CST in healthy subjects which is already slightly hyperintense on T2 and FLAIR compared to surrounding white matter. Thus, the reported characteristics observed on routine structural MRI have poor sensitivity and specificity, and conventional MRI remains only a tool to rule out diseases that mimic ALS (Filippi et al., 2010). As such, study of cerebral degeneration with imaging has progressed to advanced MRI modalities and image analysis methods such as diffusion tensor imaging (DTI), magnetic resonance spectroscopy (MRS), positron emission tomography (PET), voxel-based morphometry (VBM), and MR volumetry.

Our objective was to determine if MRI-based textures are different in ALS. Texture refers to visual patterns in images. Texture analysis uses image processing techniques and statistical methods to quantitate such patterns of voxel intensity relationships. It can capture such information as intensity homogeneity in a region, correlation of the voxel intensity value to its neighbors, and other relationships that are not appreciated by the naked eye (Kassner and Thornhill, 2010). With MR images, the methods have been successfully used to study several neurological diseases including brain tumors, (Herlidou-Même et al., 2003; Zook and Iftekharuddin, 2005) epilepsy, (Sankar et al., 2008) Alzheimer's disease, (de Oliveira et al., 2011), and multiple sclerosis (Tozer et al., 2009; Zhang et al., 2009). Robustness to MRI acquisition parameters (Mayerhoefer et al., 2009) and noise (Maani et al., 2013a,b, 2014) makes texture analysis a reliable and attractive tool for investigation of neuropsychiatric conditions. However, the majority of methods as applied to medical imaging have been restricted to 2 dimensions which has limited its utility in exploring whole-brain pathology and brain-behavioral relationships. To address this we developed a 3-dimensional texture analysis method called VGLCM-TOP-3D that performs a voxel-by-voxel statistical comparison of texture features in 3D volumetric MRI datasets (Maani et al., 2015) and applied it to study cerebral degeneration in ALS.

## Methods

### Subjects

Subjects were recruited through the University of Alberta ALS Clinic. Nineteen patients (10 males, 9 females) with clinically probable or definite sporadic ALS according to the revised El Escorial criteria (Brooks et al., 2000) were studied. Patients were sporadic (non-familial) and all had clinical evidence of UMN and LMN involvement. Patients had an average age of 56.7 ± 13.7 years (range 27–72 years) with symptom duration of 25.5 ± 16.3 months (median 20, range 9–72 months). Twenty healthy control subjects (9 males, 11 females) without neurological or psychiatric disease were included. Their average age was 56.8 ± 12.4 years (range 24–81 years). All subjects gave written informed consent, and the study was approved by the Health Research Ethics Board of the University of Alberta.

### Magnetic resonance imaging

MR images were acquired on a 1.5 Tesla system (Magnetom Sonata, Siemens Medical Systems) at the Peter S. Allen MR Centre at the University of Alberta. A conventional 3D T1-weighted MPRAGE (magnetization prepared rapid acquisition gradient) sequence (TR = 1600 ms, TE = 3.8 ms, TI = 1100 ms, voxel size 1.0 mm × 1.0 mm, 1.5 mm thick) was used to acquire coronal images of the whole brain for texture analysis. Supplementary 2D images were also acquired (sagittal T2, axial T2, coronal T2, axial FLAIR [fluid attenuated inversion recovery]). All image sequences were used in a blinded review by a neuroradiologist (DE) to classify cases as healthy controls or ALS.

### Clinical measurements

Disability was graded using the Amyotrophic Lateral Functional Rating Scale (ALSFRS). Finger tapping rates for right and left sides (FiR, FiL) were recorded as measures of UMN function (Kent-Braun et al., 1998).

### Image preprocessing

Images were normalized to the MNI152 atlas using the high-dimensional DARTEL procedure, (Ashburner, 2007) followed by correction for non-uniformity variations and intensity standardization. The preprocessing steps were performed using the VBM8 toolbox, an extension of the unified segmentation model, (Ashburner and Friston, 2005; http://dbm.neuro.uni-jena.de/vbm/) with default parameters.

### Texture analysis

Image textures are visual patterns in images defined by spatial variations in intensity. Textures not perceptible to direct visual inspection may be detected using various computational techniques, including statistical analysis. We used a popular statistical approach to texture analysis, the gray level co-occurrence matrix (GLCM) which evaluates the relationship of intensity levels between voxels in an image that are separated by a defined direction and distance (i.e., vector). The statistical information forms the basis for texture features.

Texture feature extraction was performed by the SMART (Statistical Map From Texture) toolbox using the VGLCM-TOP-3D method (Maani et al., 2015). This method calculates texture features in three orthogonal planes at each voxel, with the final texture feature value being the average of all three. The method converts an image dataset to a 3D texture map suitable for voxel-wise analysis (see Section Statistical Analysis). Default parameters (quantization level = 8, neighborhood radius = 1, distance = 1, smoothing kernel = 0) were used for GLCM computation. Four texture features were computed including autocorrelation (Auto), sum average (Savg), sum of squares variance (Sosv), and sum variance (Svar). These 4 texture features represent different statistical inter-relationships of voxel intensities and have a relationship to image characteristics. Auto is a measure of the regularity (i.e., measures the linear dependency of intensity values). A high Auto texture means high predictability of pixel relationships. Savg is a measure of intensity of texture. A higher value shows higher concurrence of high intensities. Sosv provides a measure of contrast. A higher value shows that there is a high co-occurrence between high and low intensity values. Svar is another measure of contrast. High values indicate a high concurrence among high intensity voxels and at the same time among low intensity voxels with each other. They were chosen in part based on their favorable performance in our previous work in Alzheimer's disease (Maani et al., 2015).

### Statistical analysis

Texture maps for each feature underwent voxel-wise statistical analysis using SPM8. Texture maps for patients were compared to those of controls with an *F*-test generate statistical parametric maps. A false discovery rate (FDR) at *p* < 0.05 was applied to correct for multiple comparisons. To exclude the effect of age and gender, these two factors were incorporated as covariates in the analysis. Clusters larger than 10 voxels surviving after correction for multiple comparisons were reported as regions significantly different between healthy subjects and patients with ALS. Receiver operating characteristic (ROC) curve analysis was used to calculate optimal sensitivity-specificity profiles for abnormal textures.

Regions with significant differences in texture underwent correlation analysis. This was accomplished by generating a mask for each region using the xjView Toolbox for SPM (http://www.alivelearn.net/xjview8/). The average texture values in these regions were correlated with clinical measures.

## Results

### Regions statistically different in ALS

**Figure 1:**
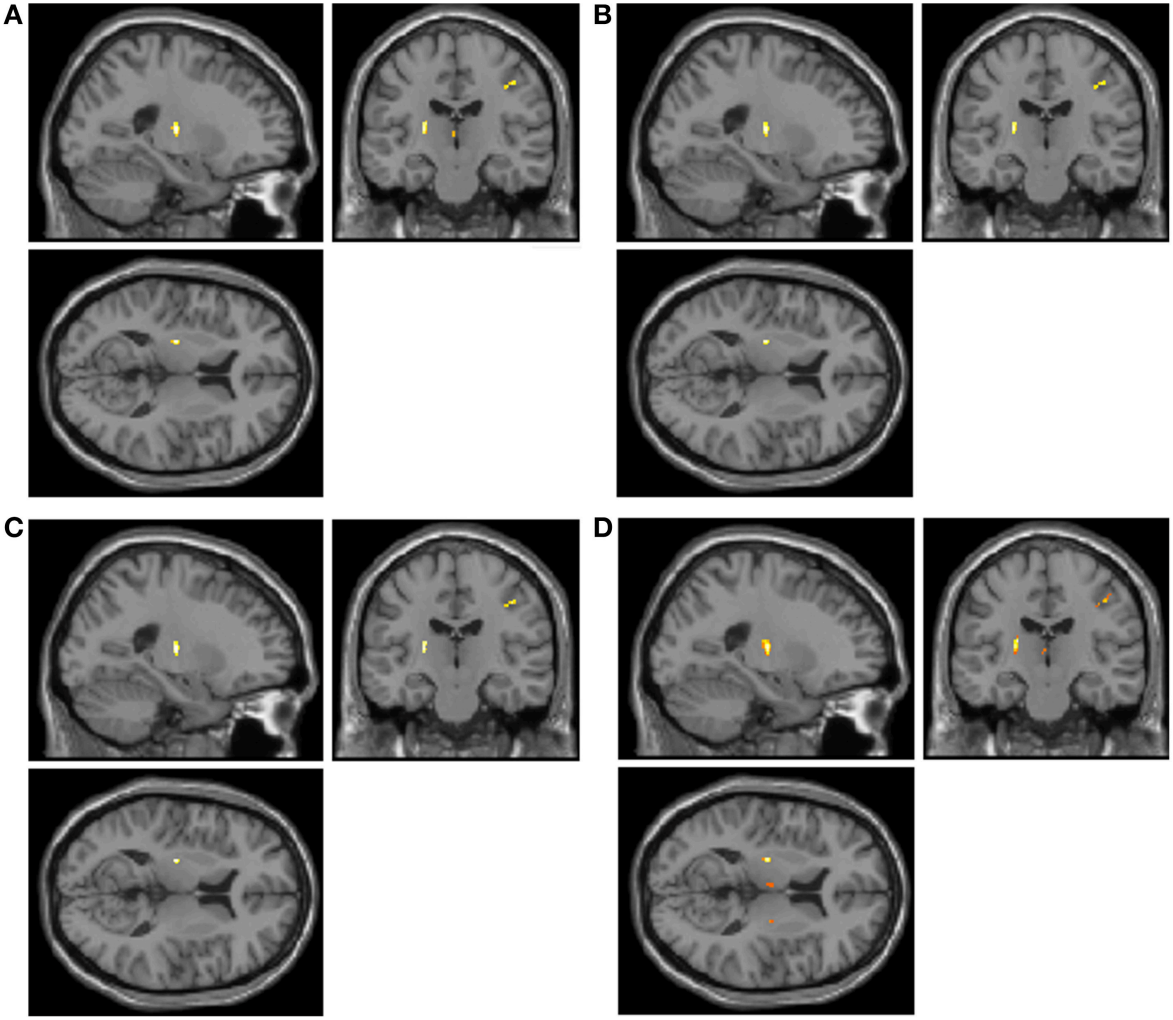
**Statistical parametric maps of texture changes overlayed on a T1-weighted image reveal differences predominantly in the precentral gyrus and corticospinal tract in all 4 texture features: autocorrelation (A, Auto), sum average (B, Savg), sum of squares variance (C, Sosv), and sum variance (D, Svar)**.

**Figure 2:**
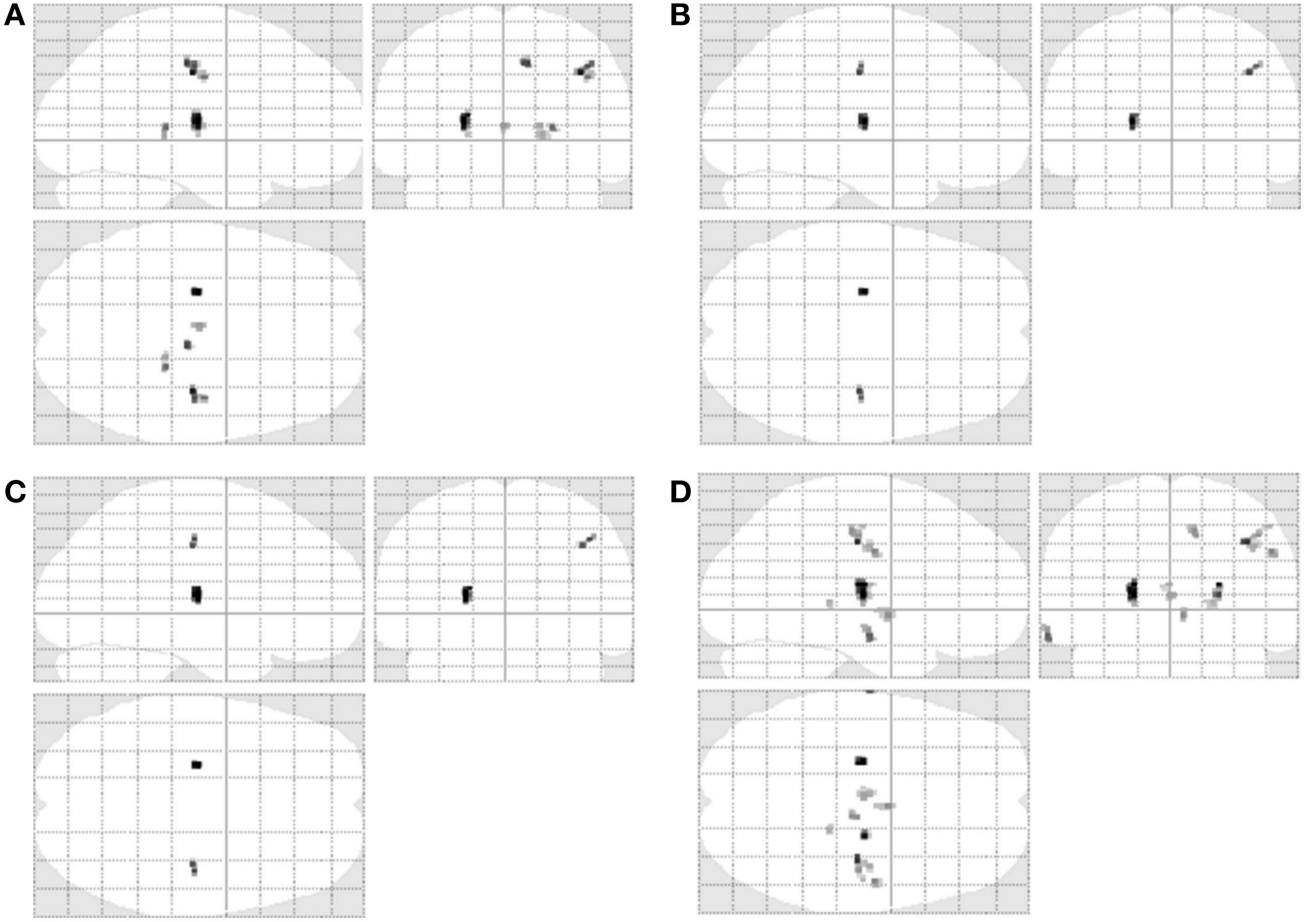
**Texture differences in ALS are presented in a glass brain representation: autocorrelation (A, Auto), sum average (B, Savg), sum of squares variance (C, Sosv), and sum variance (D, Svar)**. Changes are restricted to the right precentral gyrus and corticospinal tract for Savg and Sosv, and additionally include non-motor regions including the thalamus, temporal lobe, and cingulum for Auto and Svar.

Statistical maps revealed significant differences between the control and ALS patient groups in all four texture features (Figures 1, 2). Autoc, Savg, Sosv, and Svar were different in 7, 2, 2, and 11 regions, respectively (Table 1). Changes were seen with all texture features in the precentral gyrus and corticospinal tract (CST). Savg and Sosv differences were found only in these regions; whereas, Auto and Svar additionally were different in the thalamus, hippocampus, and cingulum. Texture values were increased in the CST and decreased in all other regions, regardless of the texture feature.

**Table 1:** **Regions showing significant texture feature differences in patients with ALS compared to healthy controls (corrected for multiple comparisons by FDR at *p* < 0.05) and their correlation with disease duration**.

**Texture**	**ID**	**Side**	**Region**	**MNI coordinates**			Texture value^*^			**ROC analysis**				**Symptom duration**	
				***X***	***Y***	***Z***	**Controls**	**ALS**	***p***	**Sens. (%)**	**Spec. (%)**	**AUC**	**Cut-Off**	***r***	***p***
Auto	1	R	TH, HC	18	−34.5	1.5	66.1 ± 4.8	58.8 ± 6.3	0.03	85	74	0.82	63.8	−0.58	0.01
	2	L	CST	−22.5	−16.5	9	84.3 ± 4.4	94.4 ± 3.8	<0.01	95	90	0.95	90.7	0.14	NS
	3	L	TH	−1.5	−15	7.5	61.4 ± 6.5	51.5 ± 9.2	0.03	95	63	0.78	52.8	−0.52	0.02
	4	R	TH, HC	22.5	−33	6	70.0 ± 4.8	62.1 ± 5.4	0.02	85	84	0.90	67.0	−0.60	<0.01
	5	R	PrG	42	−13.5	36	51.0 ± 5.2	42.4 ± 5.8	0.02	85	79	0.87	45.5	−0.59	<0.01
	6	R	PrG	37.5	−19.5	39	59.6 ± 3.5	50.1 ± 6.5	<0.01	90	79	0.89	54.8	−0.73	<0.001
	7	R	Cing, PCL	10.5	−22.5	45	56.8 ± 3.9	47.1 ± 5.0	0.01	75	100	0.95	53.8	−0.42	NS
Savg	1	L	CST	−22.5	−16.5	9	82.9 ± 4.7	94.0 ± 4.3	0.02	95	90	0.95	90.2	0.16	NS
	2	R	PrG	37.5	−19.5	39	61.5 ± 3.1	52.2 ± 6.2	0.02	95	84	0.90	57.4	−0.73	<0.001
Sosv	1	L	CST	−22.5	−16.5	9	83.2 ± 4.6	94.1 ± 4.2	<0.01	90	95	0.95	89.2	0.15	NS
	2	R	PrG	42	−18	43.5	60.8 ± 3.2	51.1 ± 6.5	0.01	95	84	0.91	56.1	−0.73	<0.001
Svar	1	L	TL	−66	−13.5	−18	44.8 ± 2.8	37.2 ± 5.3	0.01	100	79	0.91	40.1	−0.30	NS
	2	R	MB	4.5	−3	−6	67.1 ± 2.8	61.6 ± 4.6	0.01	90	79	0.85	64.4	−0.55	0.02
	3	R	TH, HC	19.5	−34.5	3	69.3 ± 3.0	64.7 ± 4.2	0.02	65	90	0.84	69.4	−0.53	0.02
	4	L	CST	−21	−16.5	13.5	85.3 ± 4.1	94.5 ± 3.7	<0.01	95	95	0.95	91.0	0.12	NS
	5	L	TH	−1.5	−15	7.5	63.9 ± 5.3	55.9 ± 7.8	0.02	90	63	0.78	57.8	−0.52	0.02
	6	R	CST	22.5	−15	13.5	85.9 ± 3.8	94.1 ± 4.6	<0.01	100	79	0.90	93.6	−0.07	NS
	7	L	TH	−1.5	−12	13.5	66.2 ± 4.9	58.1 ± 6.3	0.02	85	79	0.86	63.4	−0.41	NS
	8	R	PrG, PoG	51	−10.5	33	52.9 ± 5.5	44.2 ± 8.2	0.01	90	79	0.82	48.0	−0.44	NS
	9	R	PrG	42	−12	36	55.8 ± 4.2	48.2 ± 5.3	0.02	85	79	0.88	51.5	−0.57	0.01
	10	R	PrG	37.5	−19.5	39	68.2 ± 3.0	60.2 ± 4.8	<0.01	85	90	0.93	65.2	−0.74	<0.001
	11	R	Cing	10.5	−22.50	46.5	59.6 ± 4.2	50.4 ± 4.9	0.01	85	89	0.93	55.7	−0.41	NS

Auto, Autocorrelation; Savg, sum average; Sosv, sum of squares variance; Svar, sum variance; ID, Cluster identification number; Sens, Sensitivity; Spec, Specificity; ROC, Receiver Operating Characteristic Curve; AUC, Area Under the ROC Curve; TH, Thalamus; HC, Hippocampus; CST, Corticospinal Tract; PrG, Precentral Gyrus; PoG, Postcentral Gyrus; Cing, Cingulum; PCL, Paracentral Lobule; Temp, Temporal; MB, Midbrain;

* *(mean ± SD); NS, Not Significant*.

Texture changes had high sensitivity and specificity in discriminating ALS subjects from healthy controls (Table 1). Changes in 8 regions had 95% or greater sensitivity; 6 of these were in the motor regions (precentral gyrus or CST). Specificity was 95% or greater in 3 regions, of which 2 were the CST. There were several features which had high AUCs greater than or equal to 0.90; the best combined sensitivity-specificity profiles were seen with changes in the CST (e.g., Auto 2, Savg 1, Sosv 1, Svar 4).

In the review of the imaging by a neuroradiologist blind to diagnosis, 30 of the 39 cases (15 correctly so) were classified as being healthy controls and 9 of the 39 (4 correctly so) as ALS. Sensitivity in this classification was 21% and specificity 75%.

### Correlation with clinical measures

**Figure 3:**
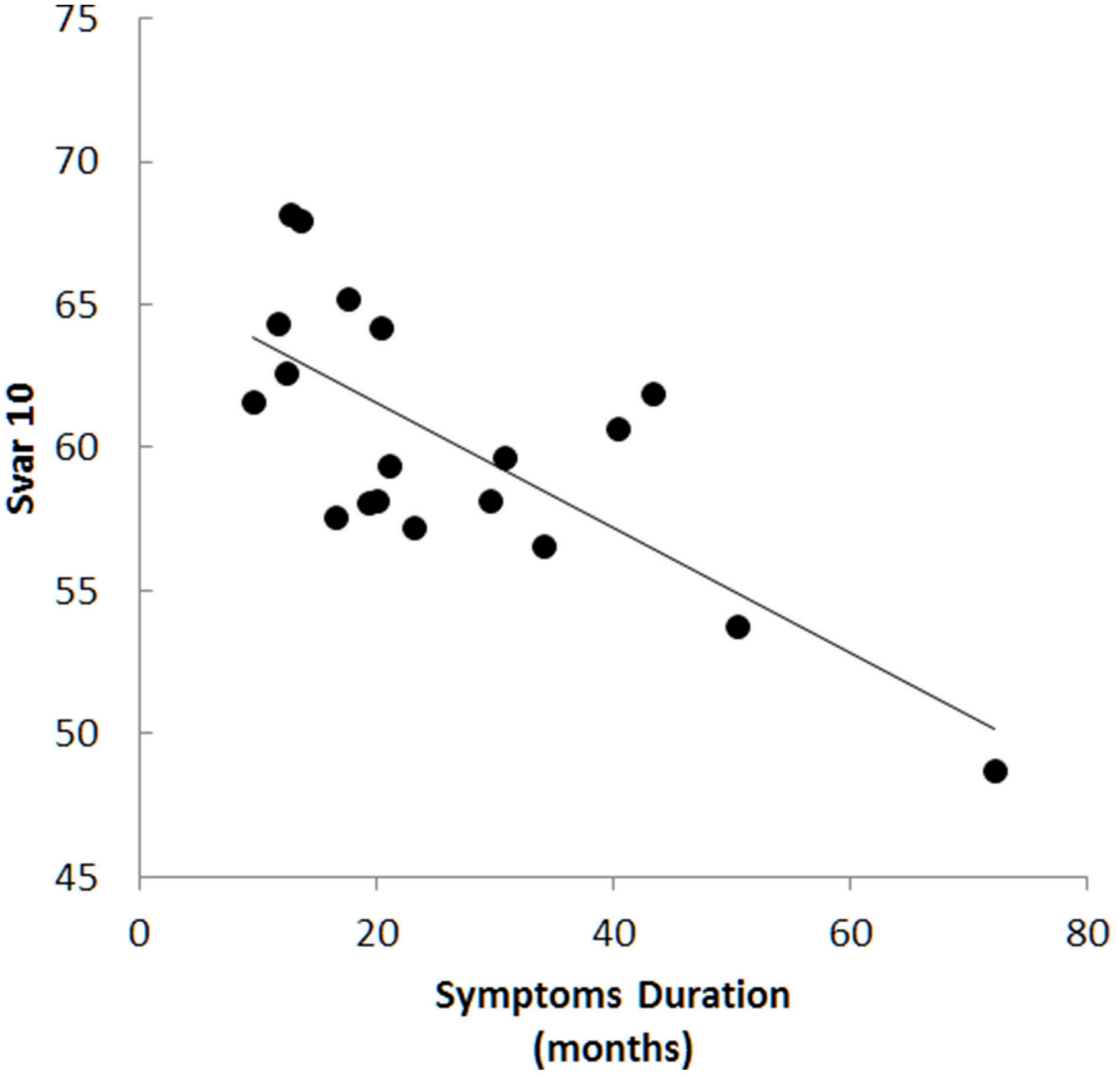
**Texture changes correlated with symptoms duration in several areas and were most prominent in the precentral gyrus, for example with sum variance in cluster 10 (Svar 10, *r* = −0.73, *p* < 0.001)**.

All texture features correlated inversely with symptoms duration (Table 1). Correlations with symptoms duration were greatest in the precentral gyrus (Figure 3), moderate in the thalamus, hippocampus and midbrain, and absent in the CST. Finger tapping on the left correlated inversely with Svar in the right CST (*r* = −0.55, *p* = 0.01). Texture changes did not correlate with ALSFRS.

## Discussion

The objective of this study was to evaluate cerebral degeneration in ALS using a novel 3D texture analysis method (VGLCM-TOP-3D; Maani et al., 2015). This method is comparable to VBM in that it performs a whole brain voxel-wise analysis in a hypothesis-free manner and generates a statistical map indicating regions different between subject groups. In contrast to VBM, this texture analysis method is not restricted to GM or WM and thus serves utility in the study of neuropsychiatric disorders, including ALS, that have pathology in both tissue classes.

Consistent with the core pathology of ALS, texture features were different in motor regions, namely the precentral gyrus and corticospinal tracts. This is concordant with changes detected using other imaging modalities (Rooney et al., 1998; Chang et al., 2005; Schoenfeld et al., 2005; Thivard et al., 2007; Iwata et al., 2008; Pyra et al., 2010; Agosta et al., 2012). Also common to other imaging studies, we found abnormalities in regions beyond the motor system, including the thalamus, (Thivard et al., 2007) hippocampus and temporal lobe, (Abrahams et al., 2004; Agosta et al., 2007; Mezzapesa et al., 2007; Canu et al., 2011; Abdulla et al., 2014) and cingulate gyrus (Abrahams et al., 2004; Grosskreutz et al., 2008; Sudharshan et al., 2011; Li et al., 2012; Hartung et al., 2014). We did not find changes in the frontal lobes, as would have been expected as frontotemporal lobar degeneration (FTLD) is the pathological substrate for the cognitive impairment observed in some patients. It is possible our patients did not have sufficient frontal lobe pathology to be detected by texture analysis; clinically they did not have frontotemporal dementia, however they had not had formal cognitive testing and mild cognitive changes due to FTLD cannot be ruled out.

The accuracy in discriminating ALS patients from healthy controls was very good in general, but did vary by region and texture feature. Many had AUCs greater than or equal to 0.90, with sensitivities or specificities of 100% for some features. AUCs and sensitivity-specificity profiles were greatest for the CST. In comparison, a neuroradiologist reviewed the imaging and classified cases blind to diagnosis. Results were of considerably lower accuracy compared to those of texture analysis with a sensitivity of 21% and specificity of 75%.

All texture features correlated with symptom duration; this was present in all regions except the CST, with the strongest association in the precentral gyrus. The biological validity of the texture feature changes is further supported by a correlation observed between Svar in the right CST with contralateral finger tapping. A correlation was not present with ALSFRS, however this is not unexpected as this disability scale is largely influenced by weakness which in ALS predominantly arises from LMN rather than UMN degeneration (Kent-Braun et al., 1998).

Since the computation of each texture feature is different, similar statistical maps reported by four different texture features suggest the findings are not spurious and reflect true cerebral pathology. However, there are patterns in the results suggesting that different textures have specificity for changes in different tissue types or are related to different pathologies. For example, although texture changes were present in the precentral gyrus and CST, the texture values were abnormal in opposite directions. Savg and Sosv changes were present only in motor regions, namely the precentral gyrus and CST and, as such, these textures features may be more specific for motor system changes and less sensitive to extra-motor phenomenon.

Furthermore correlations with symptom duration were present in the precentral gyrus and the lack of a correlation of symptom duration in the CST may suggest that changes in the CST occur very early with a subsequent floor effect, whereas motor cortex changes continue to evolve even later in the disease. Alternatively, it may be reflective of the dynamics of the texture response of different tissue types (gray vs. white matter). A longitudinal study would be required to explore this further.

A recent publication reporting texture changes in ALS used a 2D region of interest based approach (de Albuquerque et al., 2016). It calculated textures in the deep basal gray structures and the midbrain accessible in two MRI slices. Texture differences in ALS were found in the right caudate and bilateral thalami, but not putamen or cerebral peduncles. We also found changes in the thalamus but none in the basal ganglia or midbrain. In contrast to this report, our 3D method permitted a whole brain analysis and, in particular, allowed analysis of the regions where the brunt of the pathology is found (motor cortex, CST, frontotemporal lobes).

There are limitations to our study. It would have benefited from inclusion of patients with lower designations of ALS where there are fewer UMN signs (i.e., “possible ALS”), those suspected of having ALS but not meeting criteria due to absent UMN signs, and disease controls (e.g., neuropathies). Data from such sources would allow evaluation of the potential diagnostic utility of the texture analysis. Other limitations include the relatively small sample size which limited the extent to which clinical associations could be explored. In particular, future studies should have a larger sample size with accompanying neuropsychometric data to clinically validate the sensitivity of texture analysis to frontotemporal lobar degeneration. Furthermore, larger studies with more comprehensive clinical data sets would allow for a valid comparison of 3D texture analysis with conventional VBM.

In conclusion, the analysis of texture in T1-weighted MRI images of the brain revealed changes consistent with the distribution of cerebral pathology in ALS that correlated with clinical measures of cerebral dysfunction. 3D voxel-based texture analysis has potential in providing a biomarker of cerebral degeneration in ALS and other brain disorders.

## Author contributions

The authors contributed to the study in concept and design (RM, SK), analysis and interpretation (RM, YY, DE, SK), acquisition of data (SK), and overall study supervision (YY, SK).

## Funding

Financial support was provided by the ALS Association, the ALS Society of Canada, NSERC, a Queen Elizabeth II Graduate Scholarship, Alberta Innovates Graduate Student Scholarship, and Killam Memorial Scholarship.

### Conflict of interest statement

The authors declare that the research was conducted in the absence of any commercial or financial relationships that could be construed as a potential conflict of interest.
